# Exploring experiences of ableism in academia: a constructivist inquiry

**DOI:** 10.1007/s10734-021-00739-y

**Published:** 2021-07-31

**Authors:** Nicole Brown, Karen Ramlackhan

**Affiliations:** 1 Department of Culture, Communication and Media, UCL Institute of Education, University College London, 20 Bedford Way, London, WC1H 0AL UK; 2 College of Education, University of South Florida, 140 Seventh Avenue South, COQ 236A, St. Petersburg, FL 33701 USA

**Keywords:** Disability, Ableism, Lived experiences, Academics, Academia, Constructivist inquiry, Critical disability studies, Inclusion, Equity, Equality

## Abstract

To understand the experiences of the disabled in academia, a fully accessible and inclusive workshop conference was held in March 2018. Grounded in critical disability studies within a constructivist inquiry analytical approach, this article provides a contextualisation of ableism in academia garnered through creative data generation. The nuanced experiences of disabled academics in higher education as well as their collective understandings of these experiences as constructed through normalisation and able-bodiedness are presented. We show that disabled academics are marginalised and othered in academic institutions; that the neoliberalisation of higher education has created productivity expectations, which contribute to the silencing of the disabled academics’ perspectives and experiences due to constructions of normality and stigmatisation; and that it is important to enact policies, procedures, and practices that value disabled academics and bring about cultural and institutional changes in favour of equality and inclusion.

## Introduction

A wave of publications around lived experiences and theorisation of disabilities and chronic illnesses in academia (see Brown, 2021; Brown & Leigh, 2020; Dolmage, 2017; Kerschbaum et al., 2017; Price, 2011) highlights an increased interest in ableism in academia within the English-speaking countries across the world. Disability scholars whose theories of disability identify social, cultural, economic, and political dimensions (Goodley, 2014; Saltes, 2013; Shakespeare & Watson, 2001) have long questioned views of disability as representations of flawed bodies that diverge from determined norms. Yet, the pathological conceptions remain intact and detrimentally shape the higher education experiences of faculty, staff, and students with disabilities. Disability in higher education remains viewed through medicalised approaches and thus shapes how universities institute policies and practices (Merchant et al., 2019; Waterfield et al., 2018). From literature and statistics, we know that numbers of academics disclosing their specific needs are low in comparison to students or the general public disclosing their needs (Brown & Leigh, 2018). Disclosure is a complex, sensitive, and very private event that results in individuals weighing benefits and costs and identifying whether the risks may not be too great (Brown, 2020). A major challenge in disclosure of disabilities to employers relates to requests of accommodations (Lindsay et al., 2018). Employers do not readily agree to adapt organisational structures (Van Laer et al., 2020) which constructs disabled employees/faculty as inferior and characterised as deviant, unproductive, need of care (Dobusch, 2017; Mik-Meyer, 2016).

As part of her research work as well as to raise awareness of the experiences of disabled staff in academia, Nicole organised and facilitated a workshop-type conference in London entitled *Ableism in Academia* in March 2018. This conference was planned and arranged to be as accessible and inclusive as possible (for full details, see Brown et al., 2018). The purpose of the conference was to provide a space for disabled staff in academia to share experiences, whilst simultaneously allowing for data collection in the spirit of action research as a lived practice (Carson & Sumara, 1997). Theorisations of experiences alongside recommendations to make academia more inclusive have already been published (Brown, 2021; Brown et al., 2018; Brown & Leigh, 2020). This article thus explores ableism as experienced by members of the academy in the UK, through analysis of the contents from the lightning talks, the workshop posters, and feedback from the conference. The aim of this article is to provide an insight into the lived experience of ableism in academia as a stepping stone towards practical recommendations. To this end, we commence with a brief introduction into ableism and disability in higher education before outlining the details of the research design, data collection, and analysis. The nuanced experiences of disabled academics in higher education are discussed as well as their collective understandings of these experiences as constructed through normalisation and able-bodiedness. Thematic representations are explained with regard to marginalisation in academia, being silenced in academia, and perspectives on improving disabled academics’ experiences. Finally, we share implications to further research and practices to be applied at individual and institutional levels.

## Positionality, language, and terminology

Before continuing, we feel it is important to include a brief section reflecting on our positionality within our work, but also the kind of language and terminology we use. Nicole, the first author and the main conference organiser, identifies herself as a white woman of dual nationality and with a chronic condition. She is profoundly deaf and has been diagnosed with fibromyalgia, Sjogren’s syndrome, and a vertigo-condition similar to Meniere’s disease. Although Nicole *can* pass as someone with no disabilities, she uses her personal experiences with accessibility issues due to her lived experience of chronic conditions and profound hearing loss within the scope of advocacy and lobbying. Nicole’s interest in ableism is therefore simultaneously of a professional and personal nature. Karen, the second author recognises the dynamic and shifting nature of identity. She describes herself as an Indo-Caribbean immigrant woman, who experiences being othered as a result of intersectional marginalising characteristics related to race/ethnicity, socioeconomic status, gender, ability, religion, and citizenship. Karen works as a tenure track faculty member researching the marginalisation of disabled people in educational institutions. Through the critical disability studies lens, her work explores how institutional, socio-political, and cultural influences shape their experiences and trajectories.

Words, labels, terminologies, and language, in general, play an important role in communicating connotations and identity markers or in ensuring clarity in legal or policy contexts, for example. The debate around person first language is particularly contentious as using one over the other supports or denies identification with and belonging to a specific group (see Dunn & Andrews, 2015; Sinclair, 2013), and it really is up to individuals to let us know how they identify and consequently, how they would like to be addressed. Person-first language relates to description of a person with a disability, such as “person with autism”, whereas some prefer the use of language that reflects an identity-first approach, thus “an autistic person”. We have deliberately chosen not to apply the person-first language, because through our research into the lived experiences of disability, we realised that for many individuals, using the disability label is connected with activist work and raising awareness for the cause of disabled people. For the purpose of this article, we use the term disability in its broadest sense to describe the experiences of disabled persons, as well as those with chronic conditions or neurodivergences, such as dyslexia, dyspraxia, dyscalculia, attention deficit disorders, and syndromes or autism. We are fully aware that disabilities, neurodivergences, and chronic conditions are not one and the same, and yet, we do feel that in terms of ableism impacting all of those individual groups, we may take the liberty in this article to use disability to encompass them all.

## Ableism and disability in higher education

Disability studies challenge the socially constructed notion of normality and problematises the abled/non-abled dichotomy. Differences are broadly understood, accepted as natural experienced in multiple and dynamic ways and not only connected with but intrinsic to human experience (Ferguson & Nusbaum, 2012). Ableism “is a set of beliefs that guide cultural and institutional practices ascribing negative values to individuals with disabilities whilst deeming able-bodied and able-minded individuals as normal, therefore superior to their disabled counterparts” (Annamma et al., 2013:1279). In this sense, ableism is an “umbrella ism for other isms” (Wolbring, 2008:523) with disability being “cast as a diminished state of being human” (Campbell, 2001:44). Thus, bodies and minds must conform to dominant standards or become devalued and flawed. In other words, “Disabled people are the ‘able-bodied’ gone wrong” (Garland-Thomson, 1999:49). Within higher education, the organisational spaces are equally entrenched in power relations between disabled and non-disabled. A recent study illustrated the manner in which “ableist organizational space enable, and becomes dominated by, a routine employee practice that reflects and normalizes the way able-bodied employees can, and unconsciously do, interact with a space designed for them” (Van Laer et al., 2020, p. 13). The experiences of these employees in academia are lived and experienced in ways that are etched in ableist structures. Naturally, categorisations are problematic, as a group of employees within academia is not and cannot ever be considered homogenous. Yet, perceptions of otherness resulting from oppressive attitudes, policies, and practices are experienced which questions their value, independence, comfortability, and safety in this space, as is evidenced across a variety of disciplines and contexts (e.g. Bê, 2019a; Branco et al., 2019; Broderick & Lalvani, 2017; Dunn, 2019; Lynch & Macklin, 2020; Mireles, 2020; Singer & Bacon, 2020).

Research also indicates the importance of understanding the perspectives of faculty in academia in relation to disability. Moriña et al. (2020) found that disability, viewed as a valuable asset and not relegated to its established deficit position from a medicalised perspective, can be valuable in recognising that all students are important and can learn when the conditions and attitudes are properly in place. Higher education can be spaces of inclusivity and affirmative practices where disabled individuals can thrive. Disability is a judgment-based determination and the product of social, political, economic, and cultural practice (Baglieri et al., 2011). Variations from an arbitrary construction of normal become evaluations of abnormality or deviance. Across the higher education sector, the complexity of disability seems to be overlooked because of the engrained norms of normality. The concept of normal privileges some whilst marginalising others. It creates rifts and borders that function to delineate preferred characteristics (Bê, 2019b; Inckle, 2018; Pionke, 2019).

## Research approach and process

As mentioned, Nicole spearheaded a conference on the topic of ableism in academia with two aims. The first aim was to offer a conference that would be accessible for academics with disabilities and chronic illnesses to demonstrate best practice in relation to conference organisation (for full details, see Brown et al., 2018). The second aim was to use the conference as an opportunity for gathering data within the scope of a constructivist inquiry in relation to academics’ experiences of ableism in academia to develop strategies for best practices for higher education institutions. The conference therefore enabled presenters and attendees to exchange, share, and discuss experiences of being disabled and othered in academia. Constructivist, practice-based inquiries are commonly applied in educational contexts (Clarke & Erickson, 2003). As with all research, there is no one unified or right way to conduct practice-based inquiry in educational contexts, which may include action research (Sumara & Carson, 1997), reflection in action and reflection on action (Schön, 1987), case study explorations (Merriam, 1988), or living educational theory (Whitehead, 1989). What binds all these forms together is their focus on specificities of contexts. The aim of these inquiries is to use constructivism as a framework for gaining in-depth understanding of very specific, contextualised, localised circumstances that then leads to more generally applicable conclusions. Thus, practice-based inquiries make abstract, theoretical and general what is usually concrete, experiential and particular. The overarching research question for this study was “What are the lived experiences of disabled academics in contemporary higher education?”

The data collection process was organised around the two elements of the conference: peer-reviewed lightning talks and a workshop for all delegates. After a keynote presentation, the morning session entailed 13 lightning talks of approximately 5 to 10 minutes each, in which academics outlined their personal experiences of disability and ableism within academia. The topics predominantly covered experiences of invisible disabilities and neurodivergences, whereby there was a specific mention of autism, depression, microscopic colitis, grief, dyslexia, voice impairments, pain conditions, and colour blindness. Throughout the morning, the delegates were able to discuss and contribute to the lightning talks publicly and privately in plenary debates and via social media platforms, some of which were restricted to conference delegates only. The afternoon was dedicated to a workshop during which delegates considered and discussed five subsidiary questions in small groups. Groups were developed organically from the way the room had been organised in a cafe style layout, and from how individuals with particular needs would have selected their preferred seating. The small groups therefore consisted of four to six delegates, with the option of asking for a conference helper to facilitate the discussion. All groups were required to provide a summary of their discussions and responses to the questions via a poster or via email. Where required, conference helpers acted as scribes for this activity, for which flipchart paper and pens were made available. The five questions were as follows:

1. How does your disability/disadvantage affect you in the workplace and what practical effects does it have on your ability to perform your role?
2. What does your employer do to help you, and what more could they do?
3. What forum(s) is or are there at your institution for discussing matters related to ableism?
4. What forums are there in higher education to deal with these matters (from Unions to Higher Education Funding Council for England)?
5. What could/should be done to encourage members of academia to disclose their concerns/disabilities?

The conference was inextricably linked with the data collection process through interactions within the conference, through connected social media activities via the hashtag #AIA2018 and the twitter handle @AbleismAcademia. Consequently, the lightning talks, the workshop, the discussions, the recordings from the conference, the tweets, and the follow-up feedback were all considered data sources.

## Participants

This is not a typical formal study, this is an organic study, where we worked with the delegates, who all had access needs in some respect. The research participants were conference delegates. Eighty delegates attended the main conference in person, with a known 30 delegates attending a break-out event. The break-out event meant that some participants could engage in debates and discussions locally whilst watching the conference livestream and communicating with the main event via twitter and similar online platforms. All delegates seemingly had a vested personal and/or professional interest in disabilities and ableism in academia. In this sense, academics with disabilities were definitely over-represented in comparison to typical conferences or research studies. Based on the delegates’ self-identification, the male to female ratio was 1:4. Delegates were of different ages and held a variety of professional roles that ranged from doctoral scholarships, temporary part-time research or teaching positions, permanent teaching and research fellowships, and professorial and tenured positions. The main concern of all those involved in and associated with the conference related to the experience of disability itself. Many contributors and delegates felt that there was a strong need for an open debate around how disability is viewed in academia. At the same time, however, openly stating, disclosing a condition or challenge was considered problematic. Participants felt they would make themselves vulnerable, open themselves up to criticism and potential discrimination or risk to be stigmatised for being different.

## Ethical considerations

Bearing in mind the use of practice-based inquiry as an approach to the conference, institutional ethical approval for the use of the data, and the direct approaching of individuals for follow-up conversations or interviews was sought and formally granted within the specific context of the organiser actively working with participants to generate meaningful data that can inform policy and practice. Individuals provided written consent for their data and contributions to be used within the scope of a research project and dissemination via publication and further conference presentations. However, as some delegates had not disclosed their conditions or needs at their own institutions for fear of stigmatisation and discrimination (Goffman, 1990/1963), the data needed to be protected and kept as anonymous and confidential as possible. On the other hand, there were delegates who had openly disclosed their health status, who were comfortable with their disabled and chronically ill identities, who wanted to have their voices heard and who were therefore vocal in participating.

Everyone who wanted to be part of the discussions and contributed via twitter did so with the understanding that their contributions were public. If someone wanted to remain anonymous in the discussions, they had the opportunity to do so via the organised padlet, which was not included in the data analysis. Where tweets were part of the analysis, Nicole personally contacted all contributors and asked for consent to include the data.

The approach to ethical consideration was therefore focused on combining ethics of care with ethics of justice to result in a single process of “reconciliation, reciprocity, diversity and responsibility, and with an awareness of power” (Edwards & Mauthner, 2012:12). Within the context of the practice-based inquiry and in the spirit of collaborative learning during the conference, ethical approval was therefore negotiated with individuals at every step during the conference, the subsequent analysis, and write-up to offer individuals control. This flexible approach to research is not uncommon in “community-based participatory research, or emancipatory methods and methodologies” (Kara, 2018:41) and is commonly associated with methodologies developed to address various forms of oppression (Kovach, 2015:46).

## Data analysis

Qualitative data analysis is more than merely following specific steps to achieve meaning. Indeed, analysis is a craft that requires “reading beyond data” (James, 2013:574) and falling back upon “the repertoire of implicit knowledge that researchers themselves possess” (James, 2013:574). Therefore, data analysis can never be an objective process of having themes *emerge*. It is a very subjective, personal, and active process of the researcher making sense of data and pulling out specific themes and meanings (Morgan, 2018). In this project, data analysis was an iterative process of searching for and identifying themes by an active data manager who constructs themes from the data in a “transparent, reflexive and critical” way (Brown, 2019:497).

The first step was to collate all the data, the twitter feed comprising of 978 tweets from three months before the conference to three days after the event, the workshop answers on flipchart paper, the transcripts from the lightning talks, the recorded livestream, and the discussions (which totalled eight hours of footage) in Excel data spreadsheets. Thematic analysis was used (Braun & Clarke, 2006) in its intended reflexive form of research practice (Braun & Clarke, 2019) through commonly used coding practices (e.g. Bazeley, 2013; Bazeley & Jackson, 2019; Saldaña, 2009) to develop the initial codes: symptoms, management of symptoms, labelling and diagnosis, relationship with others, experiences of difference, negotiating differences, understanding of ableism, examples for improvements, and recommendations for action. These codes were then used to generate overarching themes around disabled academics’ experiences and perceptions. After all data were re-considered in connection to these bigger themes in an iterative analytical process, they were broken down again into more detailed categories in relation to descriptions of experiences, from which we formulated the resulting themes: feeling marginalised due to their perceived deficits, being silenced, and what academic organisations can do to improve their experiences as disabled people.

## Findings and discussion

In the following section, we discuss the three dominant themes in an interpretive, analytical manner with some substantiating quotes from named or unnamed delegates. With this specific approach, we honour delegates’ personal wishes regarding anonymity and protection of identities or having their quotes attributed to them in public.

## Marginalised in academia

Participants felt strongly about otherness and being othered and consistently commented on how specific ways of working or living were not accepted and acceptable in academia. For them, their lived experiences of being disabled or ill represented a “different physical reality” (a delegate), which it is necessary to make understood. Disability may result in consequences that will affect productivity and attendance. For example, negotiating everyday pressures, alongside managing a condition and soundscapes of bright rooms in busy buildings, leads to increased levels of tiredness and fatigue. One participant aptly shared,

Invisible disability in the academy is exhausting, peers & work conditions constantly overlook my needs. They have difficulty grasping fluctuations & often it’s easier to just shrug off my needs. I can so relate to Clara’s talk #AIA2018 #Spoonies

s_golightley @s_golightley

As this statement shows, otherness and othering in this context are not necessarily intentional, but are a reality of life when managing bodies and emotions. For those who have disabilities, othering and marginalisation happen because academia is built on an “assumption of a universal model of health” (a delegate), which delegates described as the inherent assumption of everybody being healthy and well.

Academics are faced with particular expectations around health, more specifically the expectation that an academic is to function able-bodied at all times. For those academics who have disabilities, the fluctuations of managing their conditions mean that they cannot meet those expectations. As a result, they are seen as less productive, underachieving, and failing in their roles as academics, which in turn others them. At the same time, when adjustments are put into place to accommodate individuals’ needs, this being treated differently is also considered a form of othering. Moreover, the stigma that is commensurate with *othering* is detrimental to the social and professional experiences of disabled persons in academia. The problems described here are due to the “inequalities, negative attitudes, misrepresentations, and institutional practices that result from the process of stigmatization” (Garland-Thomson, 1999:32). In this regard, a person is devalued because they do not fall within the normalised conception and expectations of academia. Due to their differences, perceived problematic characteristics, they are constructed as inferior to their *healthy and normal* counterparts, such as associates or colleagues.

Marginalisation in academia is even more complex, once the individual experiences of those with disabilities are concerned. These individuals are marginalised for their differences, but they also feel marginalised among an entire group of disabled academics. Delegates’ understanding of what is *normal* is so internalised that some participants not only measured themselves against the societally acceptable, standardised norms but also against internalised criteria of disability.

In absence of obvious visible identifiers (such as a wheelchair), one problematic assumption is of nondisability, leading both to individuals ‘passing’ as abled, and to unacknowledged diversity of disability present in staff and student populations. (…) It can be professionally harmful if a system either over- or underestimates the predictability of an individual’s symptoms, and so demands too much or expects too little.

Carla Finesilver, a delegate in her speech

When participants exchanged their experiences of being different and othered in the workshops and discussions, some individuals almost apologised for their issues not being “as serious as yours” (a delegate). Despite the environment and atmosphere having been one of acceptance and mutual understanding, some individuals felt invisible and hypervisible at the same time. They felt they stood out “like sore thumbs” (a delegate) for not being disabled enough for their issues and conditions to be outwardly recognisable. They felt marginalised within a group of marginalised individuals. Regardless of each’s perception of their position within the continuum of disabling conditions, the complexity and nuances of their oppressive realities in academia is the common thread which binds them together. Challenging the dominant conceptualisations of normality within institutional structures is rather difficult due to the ableist mind-set and practices that are associated with working in this context.

## Silenced in academia

Where the lived experience of disability related to academia, individuals specifically referred to the prevalent culture of overwork and productivity. Participants consistently highlighted the time and effort required for self-advocacy in order to gain access to reasonable adjustment, time and effort that other academics can spend on preparing grant proposals and publications. The burden of proof to provide necessary evidence for support lies with the individual, but at no point is there a consideration of how attending to doctors’ appointments and completing relevant forms disrupts the everyday academic routine. In the competitive, neoliberal environment of present-day academia time and effort spent on managing a condition and its effects means to lose out on important opportunities for being productive. Due to the constraints inherent in academic institutions, as a result of neoliberal ideology at work, the limited and quantified accountability measures that demonstrate productivity (i.e. publications, citations, student evaluations) often stymie the efforts of historically marginalised groups (Ramlackhan, 2019).

Academics generally are required to demonstrate their research impact and disseminate their findings, ideally to an international audience. That conferences pre-COVID-19 did not commonly allow for remote presentations, most often for financial reasons, meant that academics with disabilities were not able to attend and therefore not able to contribute to scholarly debates in that way. One of the most impactful tweets highlighting the ableist structures within academia relates to the conference organisation:

This is the first conference I have attended in 8 years.

A delegate

Live tweeting conferences is also invaluable for those of us who physically cannot make them in person! I’m eternally grateful to those who can do that so I can at least be there virtually if not in reality.

Debbie White @medievaldebbie

The ableist environment of academia, as described by participants, demonstrates that “individual bodies must conform to institutional standards, rather than restructuring the social environment to accommodate” their varying needs (Garland-Thomson, 1999:51). The capacity of individuals to conform in order to meet the required demands of working in academia is narrowly framed and constrains their ability to function appropriately and effectively. This conference was a different experience for some as it allowed them to discuss “uncomfortable truths that academia would prefer weren’t talked about” (workshop bullet point). This statement encapsulates the experience of being silenced when it comes to exploring the issues and concerns of the disabled. However, disabled academics also feel silenced in relation to their scholarly work. Academics who have openly disclosed a condition are expected to become disability activists and advocates and as such are recognised for these activities rather than their research foci (see also Brown & Leigh, 2018). As a consequence, the kinds of scholarly debates they are privy to are of specific natures and kinds, and they feel silenced as contributors to scholarly knowledge in their own right. Being perceived undesirably by only that which makes them different, such as after disclosure of a condition understood as disabling, reifies the “negative stereotypes and cultural values that surround disability…” (Vehmas & Watson, 2014:640). Furthermore, “the problem is not the disabled person; the problem is the way that normalcy is constructed to create the ‘problem’ of the disabled person” (Vehmas & Watson, 2014:640). This emphasises the importance of making apparent the entrenched assumptions of disability and disabled persons in academia in order to bring about real change and action that can shape the experiences, institutional structures, and perceptions of people within their social and professional contexts.

## Perspectives on improving disabled academics’ experiences

This theme was more prominent than others, probably because of the way that the conference specifically aimed at theorising ableism in academia and developing a strategy to improve individuals’ experiences. The key recommendations for policy and attitudinal changes therefore related to a clearer vision of inclusion in academia and more awareness through networking opportunities. On individual levels, delegates highlighted how some practices modelled in the conference need to become standard, such as live-captioning or the use of microphones, recording, and streaming. The conference was considered modelling best practices and enabled individuals to engage in academic work in an unprecedented way:

Thanks for the opportunity to attend via live stream from the sofa with a hot water bottle in E. Yorkshire #AIA2018

Kerry Pace @DiverseLearners

The overall sentiment was, however, that institutional and indeed sector-wide changes are needed to improve the experiences of academics with disabilities. Such changes, delegates commented, would only be possible with more awareness in relation to the lived experience of chronic illness and disability in academia. This is because many instances of ableism, discrimination, and marginalisation occur unintentionally but due to lack of awareness and understanding of individuals and institutions.

[We need] forums to discuss (student/staff forum, anonymous questions via whiteboard or forum, nothing really for staff except informal networks, equality/diversity committees for those who have access)

workshop bullet point

To this end, delegates highlighted, more research and activism is required. It is therein that the delegates saw the issues, because academics with a vested interest in improving the situation for disabled colleagues may not necessarily have the energy or means to pursue activism. At the same time, it was felt that education on its own would not be enough and that policies would be required to enforce cultural and attitudinal changes in the long term. Ultimately, delegates talked about moving away from what is expected as *normal* and *standard* to an environment where different forms of working and living are embraced to such an extent that “no adjustments are required” (workshop bullet point).

Delegates described the working environment of academia as male-dominated and more conducive to and appropriate for men. This negative attitude towards the working atmosphere in academia relates to the masculine, strong, competitive reinforced (Hey & Bradford, 2004) through the new managerialism in higher education (Deem, 1998, 2001; Deem & Brehony, 2005; Waitere et al., 2011). In gendered discourses of the neoliberal universities, men are engaging in real work, whereas women are forced in to lower paid or unpaid positions that involve caring and nurturing duties (Lovin, 2018). Some academics openly highlighted their scholarly and personal interest in feminism and their feminist worldviews underpinning their interpretations of what happens in academia. Others, however, who did not necessarily relate to a feminist identity, still pointed out the differences between disabled men and women, with those at the intersection of disability and femininity finding it more difficult to work and live within academia. And yet, many continue to romanticise working in academia (Lovin, 2018).

There is an important difference between visible and invisible conditions and disabilities. Depending on the kind of challenges individuals face, they may or may not have the choice to openly disclose their disabilities. This management of the self (Goffman, 1990/1963) is not always straight-forward, as sometimes tools and adjustments “out” a person for being different. And where individuals may not want their immediate colleagues or line-managers to know about specific needs and potential requirements, they may decide to withhold relevant information to avoid being “othered”. The interdisciplinary nature of the conference enables individuals to explore their experiences of ableism and safely disclose specific concerns. Individuals felt that this type of event provided the platform for discussion, away from one’s own disciplinary field and expert network. That the speakers were not surrounded by their expert colleagues, but a room full of strangers with similar experiences, seem to have made the process of disclosure more intimate, more controlled, and less daunting, even though everyone knew about the livestream and the recording being available via YouTube. The general accessibility of the conference provided a safe environment, in which “otherness” became the norm and so was not “otherness” after all. Delegates used pillows, sunglasses, blankets, and socks, for example to make themselves comfortable in the physical space of the conference room. It was acceptable to get up, lie down, remove oneself from the room, and return at any time. One delegate commented on the relevance of “awareness around people’s needs and sensitivities”.

## Conclusion

We used a Disability Studies in Education lens (Connor et al., 2008) to explore the experiences and challenges of being disabled in academia. Disability Studies in Education is an offshoot of disability studies that emphasises the importance of inclusive institutional structures, advocacy, and equitable access as well as a critique of the societal structures that have functioned to create the normal. Whilst recognising the “hegemonic ideas and practices [that] reify the ideology of normal” (Annamma et al., 2013:1280), Disability Studies in Education “argues for an examination of the institutional and cultural practices…that shape dis/ability” (Collins, 2013:284). The concept of Disability Studies in Education purports that disability “is a socially and politically constructed response to perceived difference” (Collins, 2013:284), and the deficit beliefs from dominant ableist thinking that are inherently understood and accepted as normal, for example, as found within educational environment of academia. Shifting the focus from limitations, such as attitudes, environments, practices, and beliefs, and their negative connotations (Ferguson & Nusbaum, 2012) of disabled people in academia, is a necessary step that can lead to changes in policies and processes that challenge previously conceived notions that constructs disability as negative and problematic (Werner, 2015). This type of change cannot occur with a systematic examination of the structural and cultural policies and practices that impede disabled people in their efforts to be successfully in academia. Moreover, this is not only an organisational issue but a moral and political one as well. In sum, this article’s contribution lies in (a) the elucidation of the lived experiences of ableism in academia through the application of a constructivist inquiry within the scope of the disability studies in education lens, and (b) in using this approach as a stepping stone for framing recommendations for practice and policy. With the advancement of equality, diversity, and inclusion initiatives within institutions of higher education, of which disabled academics should benefit, the following are shared to highlight a few imperatives:

Leadership matters. The power and responsibility of leaders, such as Department Chairs or Deans, are critical to creating working conditions that value and enhance individuals and to build community. These leaders are needed to institute policies, allocate funds, and provide resources in support of disabled academics. By sending clear and consistent messages of the importance of equality and inclusivity in actionable ways, these advocates will strengthen the physical space, emotional and mental well-being, and individual capacities of disabled people in higher education.

Nurturing excellence. Within the neoliberal agenda and confines of academia where incessant productivity is at the helm, disabled academics can be supported best with flexible scheduling, collaborative structures, and accessible processes. During the COVID-19 pandemic, institutions of higher education have made significant adaptations to working environments so that working from home proved to be a viable option to the traditional office work space. Although not everyone has benefitted from working from home, these changes have proven to increase productivity for disabled people. In March 2018, the Ableism in Academia conference was one of its kind because of its flexibility, inclusivity, and accessibility offering disabled academics a unique opportunity to engage in knowledge exchange and research dissemination. Flexible and adaptable processes and procedures need to continue if we are to maximise their strengths and excellence. For example, trainings or professional development for leadership personnel, academics, and staff focused on understanding disabled experiences and ways to support them is needed. Individuals working in higher education contexts with disabled academics first must acknowledge their views and perspectives about disability and configure ways to support colleagues and co-workers that value and enable their contributions.

Above all, therefore, an attitudinal shift is required that will allow for disabled people to engage in and with higher education as a matter of course. Whilst we aim towards a Universal Design for Learning to support disabled students (Bracken & Novak, 2019), a more ubiquitous universal design that truly focuses on inclusion and equality rather than reasonable adjustments would benefit everyone working, studying, living, and breathing the academy. An attitudinal shift is also required for an improved understanding of *how* disabled faculty, staff, and students engage in and with academia. Within disability studies and ableism studies, there is a strong narrative relating to the disability experiences and discourses of who is and should be able and allowed to research and report on disabilities. The slogan “nothing about us without us” comes to mind (e.g. Bryden, 2015; Charlton, 2000; Yeo & Moore, 2003). However, research into academic’s lived experiences of ableism and disabilities is complex. On the one hand, the involvement and participation of those directly affected is needed; on the other hand, experiences of marginalisation and othering need to be navigated alongside stigma and matters of disclosure. Hence, there is significant tension between the conflicting need of disclosing to be involved in relevant research and of not-disclosing to maintain one’s existing public, scholarly identity. As we have seen from the findings section, within the context of higher education, academics are keen to be involved in scholarly debates and to engage in activist work, but not at the expense of their career trajectories and potential opportunities to pass as non-disabled.

As with all research, this report should not be considered an end-point, but as a resource to draw on for future work. Considering our own conclusions, we have identified three main avenues for further research: First, the practical recommendations from this article alongside previously published work could be used for the development of equality, diversity, and inclusion training, which should then be evaluated for its effectiveness in raising awareness, changing attitudes, and creating long-term change. Second, we see an important connection to be made between the existing work around ableism in academia and the most recent developments around flexible, different, and creative ways of working that have arisen during the COVID-19 pandemic. And third, building on the constructivist inquiry applied in this project, there is a real opportunity here through and evaluation study to contribute to methodological advancement within the scope of participatory, democratic ways of working for data generation and analysis. The framework of the constructivist inquiry applied during the conference enabled individuals to be part of the conversation and shape the discourses, whilst simultaneously remain anonymous, if they so wished. The research here was not done *to*, *on*, or *with* them, but very much *by* them. The result is a disabled-consciousness conceptual approach (Ramlackhan, 2021) that frames ways of thinking, being, and doing within the professional spaces of academia and that outlines how to construct these spaces with a disabled-conscious mind-set. It is this kind of approach that enables and empowers and that as individuals and institutions we need to strive for. We all need to become more aware and conscious of what it means to be disabled in higher education and of how we can be allies in that environment. The main recommendation resulting from our work therefore is learning to listen.
